# Explainable AI for Bearing Fault Prognosis Using Deep Learning Techniques

**DOI:** 10.3390/mi13091471

**Published:** 2022-09-05

**Authors:** Deva Chaitanya Sanakkayala, Vijayakumar Varadarajan, Namya Kumar, Karan, Girija Soni, Pooja Kamat, Satish Kumar, Shruti Patil, Ketan Kotecha

**Affiliations:** 1 Symbiosis Institute of Technology, Symbiosis International (Deemed) University, Pune 412115, India; 2 School of NUOVOS, Ajeenkya DY Patil University, Pune 412105, India; 3 School of Computer Science and Engineering, University of New South Wales, Sydney, NSW 2052, Australia; 4 Symbiosis Centre for Applied Artificial Intelligence, Faculty of Engineering, Symbiosis International (Deemed) University, Pune 412115, India

**Keywords:** spectrogram, convolutional neural network, anomaly detection, remaining useful life prediction, VGG16, LIME analysis

## Abstract

Predicting bearing failures is a vital component of machine health monitoring since bearings are essential parts of rotary machines, particularly large motor machines. In addition, determining the degree of bearing degeneration will aid firms in scheduling maintenance. Maintenance engineers may be gradually supplanted by an automated detection technique in identifying motor issues as improvements in the extraction of useful information from vibration signals are made. State-of-the-art deep learning approaches, in particular, have made a considerable contribution to automatic defect identification. Under variable shaft speed, this research presents a novel approach for identifying bearing defects and their amount of degradation. In the proposed approach, vibration signals are represented by spectrograms, and deep learning methods are applied via pre-processing with the short-time Fourier transform (STFT). A convolutional neural network (CNN), VGG16, is then used to extract features and classify health status. After this, RUL prediction is carried out with the use of regression. Explainable AI using LIME was used to identify the part of the image used by the CNN algorithm to give the output. Our proposed method was able to achieve very high accuracy and robustness for bearing faults, according to numerous experiments.

## 1. Introduction

**Figure 1:**
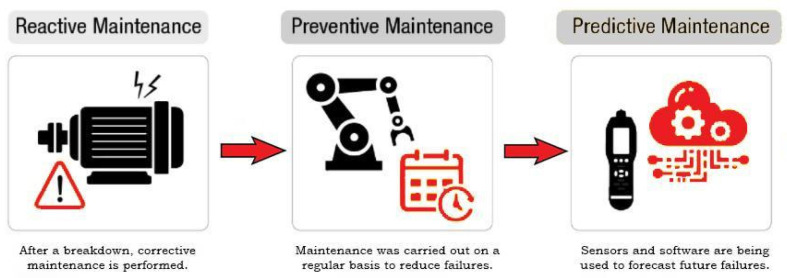
Evolution of maintenance.

Predictive maintenance is a new technique in mechanical engineering that is attracting interest from both academics and the industry. Because unexpected breakdowns can result in misplaced production, failed transportation systems, and poor client reviews, it is critical to implement this method, which provides users with an integrated view of a machine’s or device’s health status and is expected to reduce unnecessary preservation or maintenance while also improving the machine’s reliability and safety. After a fault has been diagnosed, an accurate estimate of the remaining useful life (RUL) becomes critical for predictive maintenance deployment. Once a fault has occurred, the RUL estimation must be used to determine when maintenance steps should be taken to avoid catastrophic failures. Mechanical constructions, in the real world, often degrade over time rather than failing suddenly. As a result, an ideal fitness indicator is often constructed first to indicate the propensity of fault growth over the course of a system’s lifespan, and the RUL prediction is mostly dependent on the established fitness indicator via proprietary algorithms [1]. The following Figure 1 shows the evolution of maintenance of machinery.

The growth of the industry and the benefits of updating industries to Industry 4.0 and predictive maintenance can easily be seen in the given data (“Maintenance Statistics: Predictive & Preventive, Labour & Costs”):

- The graph of usage for predictive maintenance went from 47% in 2017 to 51% in 2018, although preventive maintenance remains the most popular among 80% of maintenance personnel;
- 80% of production factories use preventive maintenance, and a majority of them use predictive maintenance with analytical tools;
- Predictive analytics yields a greater return on investment, and it leads to savings of 30% to 40%. This shows that the industry has been making a large profit after the implementation of predictive maintenance;
- As of 2018, the foremost common challenge facilities face is a lack of resources, together with human, technical, and strategic resources. Eventually, total productive maintenance has been shown to extend plant capability by over 10% and productivity by 50%;
- More than 30% of all the deaths in a manufacturing unit are associated with maintenance activity. The use of predictive maintenance can help us decrease that number;
- Poor maintenance techniques can lessen a company’s manufacturing capability with the aid of using as much as 20% [2,3,4,5,6].

This paper mainly focuses on the maintenance of any machine that generates vibration. For rotating machinery, a rolling bearing is considered to be an important part, and its working influences the machinery’s health directly [7]. As a result, it is necessary to keep track of its status in order to avoid production losses or maybe fatalities as a result of accidents [8]. Estimating RUL accurately for rolling bearings promotes production safety and saves significant amounts of money. The initial step of the process will be gathering the data from the machinery in the form of vibration or temperature with the help of different sensors such as thermal, acoustics, etc. the next step is processing these data using FFT (fast Fourier transformation) methods such as time or frequency domain, or both even the time-frequency-domain analysis and after which the data set will be split into training and testing dataset. In the next step, the data are passed to suitable AI algorithms for fault detection and RUL prediction.

Some of the previous works on predictive analysis or predictive maintenance show how different algorithms have given stable results with different kinds of data (acoustic, vibrational, thermal, etc.) [9]. Cao et al. propose a domain adaption-based CNN model for machine fault transfer diagnosis from a constant speed state to a speed state that varies over time [10]. Kamat et al. propose an anomaly-onset aware framework for anomaly timestamp identification on the basis of time-domain characteristics [11]. Binayak Bhandari presents a study of popular deep learning models for spectrogram-based audio feature extraction to classify machine roughness [12]. Feng He and Qing Ye propose the spectrogram-based fault diagnosis method for bearings using the wavelet packet transform and CNN technique on the CWRU bearings dataset [13]. 

Before concluding the final methodology for the whole process, we reviewed some of the key findings of the recent development in predictive maintenance. To begin with, we understood that fault detection becomes very easy to implement using low cost and high accuracy once the vibration signal is combined with ML techniques. This leads us to the selection of our dataset, which is a bearing dataset containing its vibrational data. The anomaly prediction is added based totally on the subdivision and extension to RUL to explain and predict the device situation at the healthy stage, which fills the space of remaining life studies absence [14]. STFT may be a great tool for evaluating bearing signals under difficult situations or with background noise, thanks to the benefits of frequency-domain analysis for nonstationary indicators [15]. After implementing FFT, we were able to convert the vibrational data into spectrograms using this method. Deep learning algorithms that have been trained on massive amounts of training data are often used to learn complex models. RUL is a linearly decreasing characteristic that approaches zero as the device approaches failure. On the other hand, RUL estimation cannot be adequately predicted before the onset of machine deterioration. Many other studies address this issue by defining a maximum RUL level and claiming that RUL above that level is constant [16]. Following the investigation, it was discovered that insufficient research had been conducted to provide a consistent framework for anomaly detection and RUL prediction. Throughout the wear-out era, the majority of bearing research has focused on RUL calculations [17,18]. Detecting the bearing degeneration is not mentioned in many of the techniques. Estimation of the RUL when the bearings are in suitable condition is very difficult; therefore, detecting the first signs of degradation is an efficient way to predict a malfunction. Many of these procedures necessitate large data in order to recognize different examples and accurately predict the RUL.

The construction of an effective model for estimating RUL is problematic due to variability in deterioration curves and physical features of bearing systems. Using data-driven techniques, sensor data are translated into a stochastic or non-stochastic correlation model [15]. Understanding the deterioration process is not that important; rather, enough past-life data are required.

One of the most challenging parts of developing a deep learning model for RUL estimation is identifying the reference truth RUL for the acquired data. RUL is a linearly decreasing function that approaches zero as the machine approaches failure [19]. The RUL estimate, on the other hand, is not clearly specified before degradation begins. Most studies address this issue by establishing a maximum RUL level and assuming that RUL remains constant after that point [12]. Furthermore, choosing the best RUL is subjective, and the appropriate value for many systems is rarely the same. There has not been much study carried out to provide a consistent methodology for identifying anomalies and forecasting RUL as soon as they happen.

The novelty of this research paper is the combined framework for RUL prediction and fault detection. Most of the research in the past has worked on time-domain characteristics of vibration data for fault diagnosis and prognosis separately. To the best of our knowledge, not much work has been carried out by converting vibration data into spectrograms for fault detection and further prognosis. A spectrogram reveals numerous and nuanced indications of damage. Unusual bands in spectrograms can provide extremely useful information regarding future damage. To anticipate the failure of a device, time-domain analysis restricts us to using only the characteristics related to the time the device is operating or not working. The spectrogram, on the other hand, depicts patterns of energy shift and provides a clearer picture of how vibrations vary over time. So basically, the flow of the process goes as follows: first, we convert that raw vibration data into a spectrogram and hence apply clustering on it to split the spectrogram dataset into normal and abnormal spectrograms. This spectrogram dataset is passed to a CNN model for the fault diagnosis. After the fault is detected, RUL prediction is carried out by passing the dataset into a regression algorithm.

This study makes the following contributions:

- Spectrogram generation: this paper focuses on the use of spectrograms for fault detection. The raw vibrational data from the bearing dataset were converted into spectrograms after FFT analysis, which further affected the accuracy of the model in a positive way;
- Using a clustering algorithm to divide the spectrogram dataset into two parts, i.e., normal and abnormal;
- Using CNN VGG16 for fault detection through the spectrogram dataset, 16 convolutional layers helped us to achieve better accuracy;
- Explainable AI is used to explain what part of the spectrogram image was used by the CNN model to predict the required results;
- Finally, the RUL prediction and fault detection on one single framework.

## 2. Dataset Description

The employed IMS dataset in the study refers to the “test-to-failure” life of four bearings [17] which were placed on a shaft that was then rotated with a constant speed of 2000 RPM using an AC motor. Using a spring apparatus, 6000 lbs. of the radial load was applied to the complete setup. The bearings are made to operate until failure, which majorly occurred after they exceeded their designed lifetime of 100 million revolutions.

The complete data packet (IMS bearing data zip file) consists of three datasets, namely Test 1, Test 2, and Test 3, which describe the test-to-failure experiments performed on the bearings. As shown in Table 1 These datasets are basically individual files that contain snapshots of 1 s vibration signal snapshots, which were documented at predetermined interims. With a sampling rate set of 20,000 Hz, each file has 20,480 data points, which were recorded in both horizontal and vertical directions for Test 1 and in only horizontal directions for Test 2 and Test 3. To ensure the model receives sufficient normal as well as abnormal data for training, the study makes use of two out of the three datasets. 

The challenge addressed in this study is to develop a complete framework that assesses the health state of the equipment (state of health—SoH), detects faults in the equipment, performs outlier analysis, and predicts the remaining useful life (RUL) of equipment. 

## 3. Methodology

**Figure 2:**
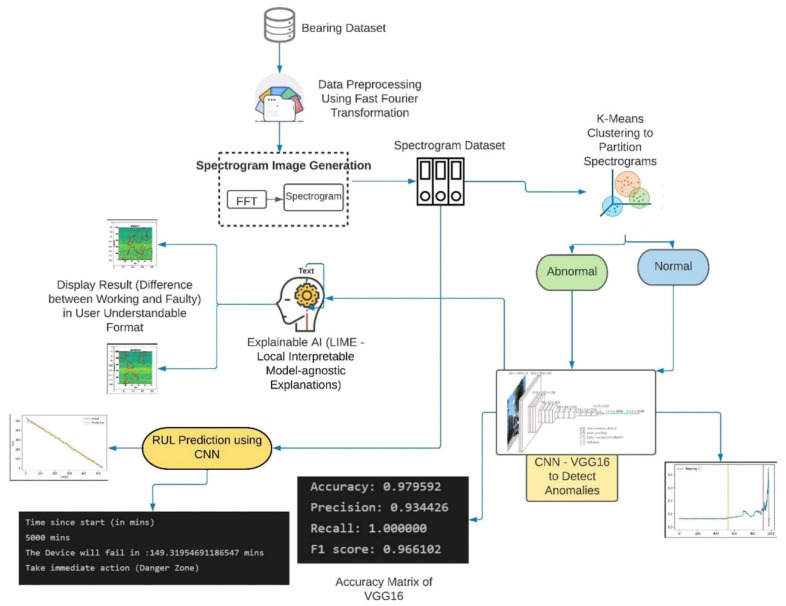
Proposed methodology diagram.

Figure 2 describes the proposed irregularity-encounter informed remaining useful life prediction using the spectrograms framework. The research is unique in that it uses spectrograms to build a unified plan of action for fault identification and RUL forecasting. To identify the source of anomalies and estimate RUL, the framework employs unsupervised clustering and supervised deep learning approaches. There are 5 phases to the framework: (i) data pre-processing and feature engineering; (ii) spectrogram generation and unsupervised clustering for condition monitoring data; (iii) supervised fault detection for fault prognostics; (iv) explainable AI to provide findings in user-understandable format; and (v) RUL prediction for fault prognostics. The following are detailed descriptions of each of the phases represented in Figure 2:

### 3.1. Data Pre-Processing and Feature Engineering

The variation in vibration data can give prominent signs about the severity and, in turn, the nature of the problem, which could then be used to predict the remaining useful life of the machinery. On approaching the onset of degradation of the bearings, the analysis performed on the change in amplitude and distribution can bring forth the possibility of early fault detection. The data points captured in a specific time period can be represented by a single vibration signal. In the scenario presented by the IMS dataset, 20,480 data points that were captured after an interval of 10 min are to be treated as a single signal, and it can be observed that as the disparity in the amplitude levels of these points increases, the severity of fault increases and with that, the signals’ mean and standard deviation that indicate the distribution of the signal display change as well.

Features of any raw sensor data have proven to give more insights into the health state of the bearings; hence, 22 major time-domain features that help define machinery failures, such as RMS, peak, skewness, and others, were extracted, and the bearing data were then visualized.

**Figure 3:**
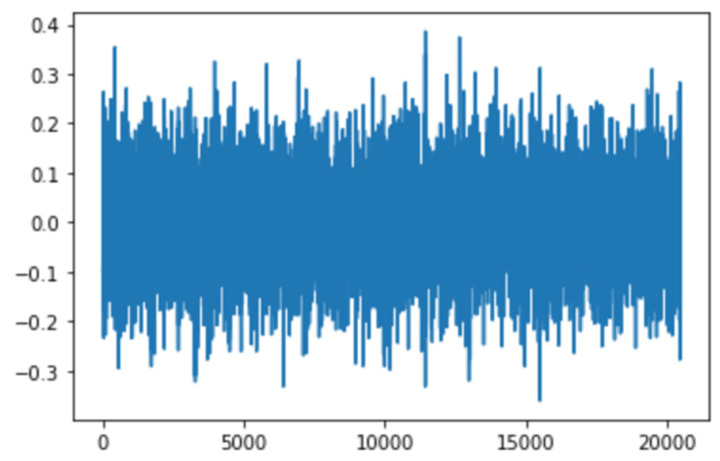
Vibrational signal at 10:32:39 on 12 February 2004.

After visualizing the bearing data in the time domain, as seen in Figure 3, and performing feature engineering, we then proceed to generating spectrograms.

### 3.2. Spectrogram Generation and Unsupervised Clustering for Condition Monitoring Data

At the end of Phase 1, after visualizing the bearing data, we move toward the time-frequency domain using spectrograms. Spectrograms can be briefly explained as the visual representations of frequencies of a given signal with time [20]. In spectrograms, one axis represents time, while the other represents frequency, and the magnitude of the frequency at a given time is represented by the color. A spectrogram basically depicts the strength of a signal over time at various frequencies in a waveform [21]. 

The decision to move toward making use of spectrograms instead of moving forward in the time domain was taken because a time-domain analysis can detect a flaw in a device, but it cannot pinpoint its location or nature. “As a collection of time-frequency analyses, the spectrogram can be used to identify characteristics of nonstationary or nonlinear signals. For this reason, a spectrogram is a helpful tool for analyzing real-world data where there are various frequency components and/or mechanical and electrical noise.” [22]. There are numerous and intricate signs of damage in a spectrogram. Atypical bands can, nonetheless, provide incredibly helpful information about future damage. Time-domain analysis limits us to use only the features related to the time of the device working or not to predict the failure of the device. The spectrogram, on the other hand, illustrates patterns of energy change and gives us a better look into how the vibrations change over time. The spectrogram is a combination of time-frequency analyses can be used to discover nonstationary or nonlinear properties. As a result of this, a spectrogram is a useful tool for assessing real-world data with several frequency components as well as mechanical and electrical noise [23].

In a dynamic environment, a spectrogram is extremely useful for vibration analysis. It depicts energy change patterns that may not be seen in an FFT. A spectrogram, as opposed to an FFT, which is the representation of the signal in the frequency domain [24], provides a more detailed view of how the vibration evolves over time.

There are various indications in a spectrogram, which can be complicated at times, but the spectrograms can still provide extremely useful information about prospective harm [25]. Hence, the vibration data were changed from the time domain to the time-frequency domain, which was implemented by first moving to the frequency domain using fast Fourier transformation. This was performed by dividing the time-domain signal into shorter segments of equal length and then applying FFT to each segment. The spectrogram is then a plot on each segment. 

To move from time domain to frequency domain, the discrete Fourier transform of each signal, which is a sequence of data points in the dataset, was computed using the fast Fourier transform algorithm. The fast Fourier transform algorithm is able to convert a signal into its respective individual spectral components and thereby gives us information about the signal pattern. The following equation results in the Fourier transform of a time series *x_t_* for frequency f cycles per *n* observations, where *f* can take values such as 0, …, *n* − 1.

(1) $$z_{p}=\sum_{t=0}^{n-1}x_{t}exp\left(\frac{-2\pi ift}{n}\right)$$

It can be inferred that the result is a complex number, *z_p_* in which the real part takes the place of the cosine variation, and the imaginary part takes the place of the sine variation, which is in accordance with the Euler’s relation

(2) exp(iθ)=cosθ+isinθ

The next step is generating spectrograms that are said to be time-domain representations and can calculate the frequency content of the signal over a short time span and display it as the frequency spectrum as a function of time. Signals with time-varying frequency content, such as the data used in the study, present vital information.

**Figure 4:**
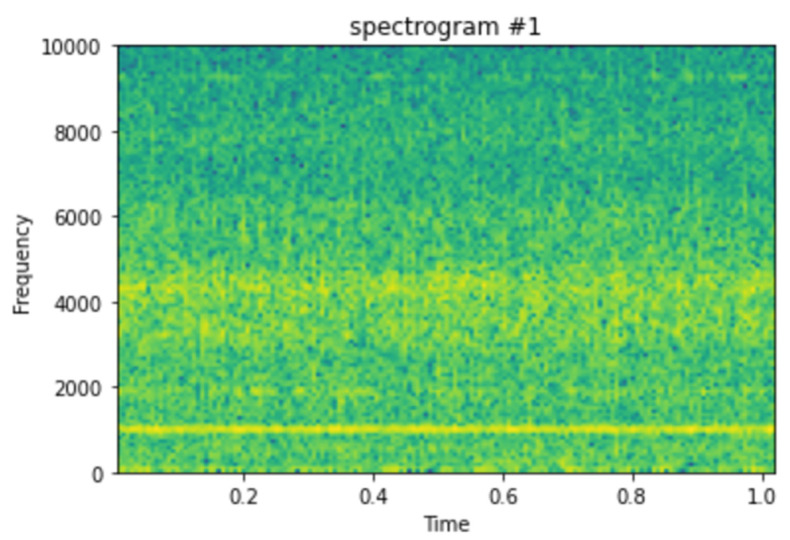
Spectrogram generated for vibrational signal at 10:32:39 on 12 February 2004.

The spectrogram will be generated by calculating short Fourier transforms on consecutive sections of the signal and presenting them as the signal’s frequency content over the time period. A spectrogram, in general, aids in the visualization of changes in signal frequencies over time, with amplitude depicted in the third dimension with changeable brightness or color, as stated before and as can be seen in Figure 4.

#### Spectrogram Generation

The spectrograms were generated on the 984 vibrational snapshots taken at 10 min intervals. For generating the spectrograms, we have taken each snapshot from the dataset, which contains 20,480 vibrational data points. Figure 4 depicts a single vibrational signal snapshot taken at 10:32:39. This is the first vibrational signal from our dataset, which is the start of recording the vibrational signals.

The spectrum of each section is generated once the data points are separated into NFFT length segments. Each segment is given the windowing function window, and the amount of overlap between segments is set with overlap. The spectrogram is represented graphically as a color map (using imshow).

Figure 4 shows the spectrogram generated for the first vibrational signal, and the sampling rate for the signal is 20,000 kHz. The third dimension, color, is used to describe the amplitude (or power or “loudness”) of a single frequency at a specific period, with dark blues equating to low amplitudes and brighter hues up through red corresponding to regularly more strong (or louder) amplitudes. The yellow markings on the image show the potent/high amplitudes.

**Figure 5:**
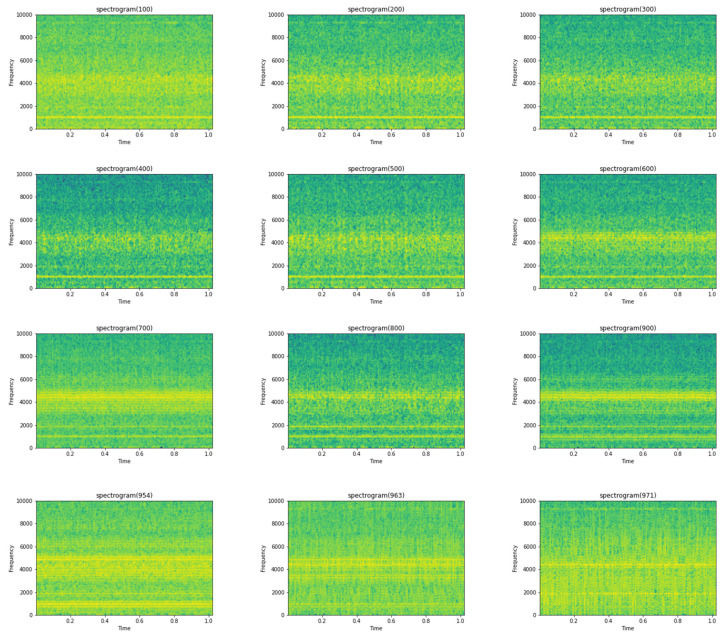
Sample spectrogram images at various timestamps.

Figure 5 shows the spectrogram images of the vibrational signals taken at multiple timestamps. We can notice here that as time passes, the amplitude of the signals is increasing, which is denoted by the yellow markings on the spectrogram.

Lastly, the final stage of Phase 2, which is partitioning the spectrograms into normal and abnormal datasets using the K-means clustering algorithm, is implemented. K-means clustering is an unsupervised ML algorithm that helps solve clustering problems when dealing with unlabeled data that, in the case of the study, are the spectrograms generated before. Here, the method was used to form 2 clusters, namely, normal and abnormal. The algorithm, K-means clustering works in the following way—(i) decision about the value of k is taken, which in the case of the study was taken to be as 2; (ii) random k centroids are selected; (iii) each data node is assigned to their closest centroid, which later forms the necessary clusters; (iv) variance is calculated, and a new centroid is placed for each cluster that was formed; (v) the first 3 steps are performed repeatedly and, lastly, after the necessary reassignments, the model is deemed as ready [26,27,28]. The result of the K-means clustering model after performing the above-mentioned steps as well as the distribution of the dataset, which can be observed in the following section.

Though the algorithm helped with the labeling of the spectrograms as normal and abnormal, it single-handedly cannot be used to detect faults, and hence, the study moves toward the next phase, which is the implementation of the proposed VGG-16 model [15].

### 3.3. Supervised Fault Detection for Fault Diagnosis

Anomaly detection refers to identifying abnormalities in otherwise normal data. Detecting anomalies in the machinery is the first step toward any successful efforts made in the field of predictive maintenance. Furthermore, machine health monitoring can be aided as well through these initial efforts [29]. The proposed methodology works toward identifying the exact timestamp at which the first fault occurred and thus helps the study by giving insights into the RUL of the bearings. 

Phase 3 makes use of VGG 16 for fault detection, which is a pretrained convolutional neural network with a depth of 16 layers. The pretrained model has a huge variety of categories and hence was decided on for the fault detection stage. The network has knowledge of rich feature representations for an immense variety of images with an input size of only 224 by 224 RGB.

The proposed model can be summarized as follows:

- The model created was sequential in nature, which meant that all layers of the model were arranged in a sequence;
- Since the model is highly powerful, the only pre-processing it performs is the subtraction of RGB values from each of the pixels;
- The image is then passed through a series of convolutional layers, each of which has filters with a 3 by 3 receptive field;
- The convolutional strides are predetermined to 1 pixel only;
- The spatial padding of the convolutional layer input is 1 pixel for 3 by 3 convolutional layers, ensuring that the spatial resolution is maintained even after convolution;
- The 16 convolutional layers are followed by a portion of the 5 max-pooling layers, which perform spatial pooling over a 2 by 2 pixel window with a stride of 2;
- Three fully connected (FC) layers are then added on top of the stack of convolutional layers, the first and second of which have 4096 channels each, and the third of which has 1000 channels;
- The last is the soft-max layer, and the hidden layers of the model are all equipped with ReLU non-linearity;
- The model uses an Adam optimizer to accelerate the gradient descent algorithm by considering the exponentially weighted average. Adam optimizer inherits the strengths or the positive attributes of both momentum and root mean square propagation (RMSP) and builds upon them to give a more optimized gradient descent. This helps the algorithm converge to the minima at a faster pace. Building upon the strengths of previous models, Adam optimizer gives much higher performance than the previously used ones and outperforms them by a big margin, giving an optimized gradient descent.

**Figure 6:**
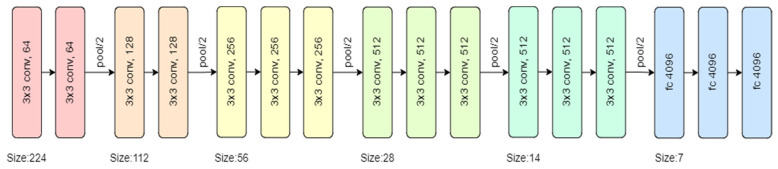
VGG-16 model architecture.

The visual representation of the proposed VGG-16 model can be seen in Figure 6.

### 3.4. Explainable AI to Generate Results in User-Understandable Format

To understand the results of our fault detection model, the proposed methodology includes the use of the explainable AI technique, LIME (abbreviation for local interpretable model-agnostic explanations). LIME attempts to obtain an understanding of the model by creating a disturbance in the input images that were fed to the fault detection model and observing how the predictions of the model differ.

The way LIME works is by manipulating the input data, which for the study was the spectrograms, and creating a sequence of false data that partly contains the original features. Hence, for the model, different versions of the input spectrograms were created, and a definite amount of randomly selected sections, that is, pixels of the spectrogram, were removed. These false data were then assigned to either the normal or the abnormal category. 

**Figure 7:**
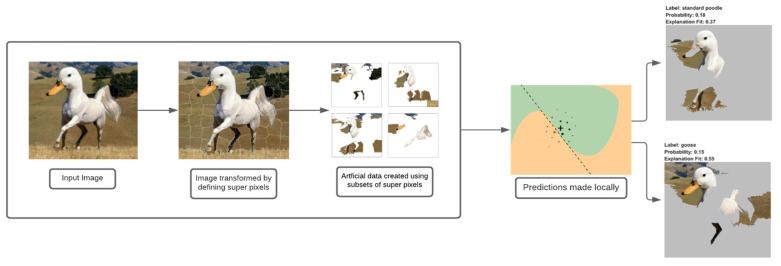
LIME working.

The visual representation of LIME can be seen in Figure 7.

The LIME map is generated for the fault detection model’s predictions, where the image that was predicted as normal has a region highlighted in blue, which is the part used by the model to predict it as so, and the image that was predicted as abnormal has a region highlighted in red for similar reasons.

### 3.5. RUL Prediction for Fault Prognosis

**Figure 8:**
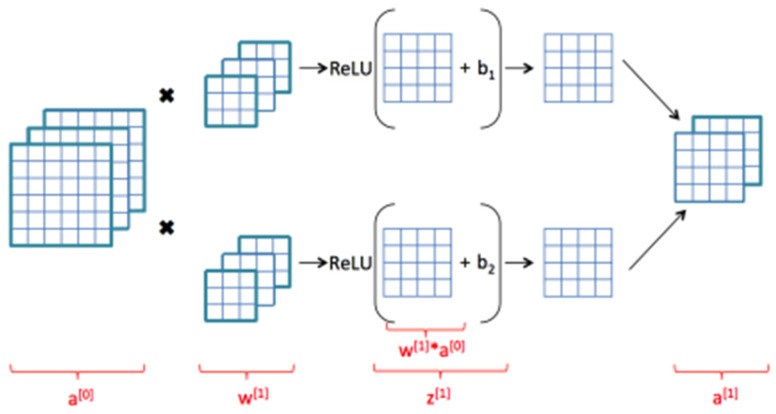
Convolutional neural layer. [0] and [1] represents the first and second neurons of the layer, * represents multiplication and x shows the conversion of the image into smaller parts in the next layer.

The proposed methodology includes the use of the time-domain features that were derived in earlier stages along with a simple CNN model with two 2-D convolutional layers. Sliding convolutional filters are applied to a 2-D input in these layers. The layers convolute the input by first moving the filters vertically and horizontally along it, then computing the dot product of the weights and the input, and then adding a bias factor [30,31,32,33,34,35,36]. One such layer can be found in Figure 8.

**Figure 9:**
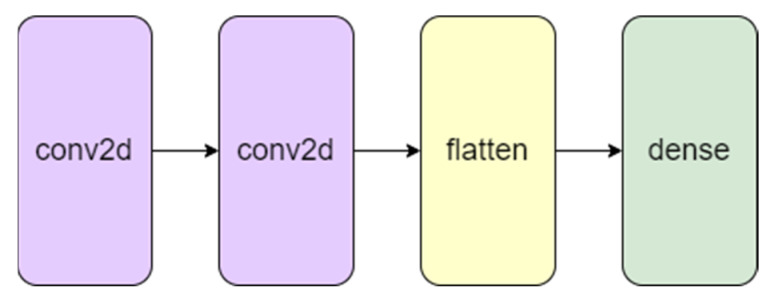
Visual representation of the proposed CNN model for RUL prediction.

CNN has proven time and time again to be a powerful tool for prediction and hence was decided on as the final key to the framework [37,38,39]. A visual representation of the proposed CNN model can be found in Figure 9.

## 4. Results

**Figure 10:**
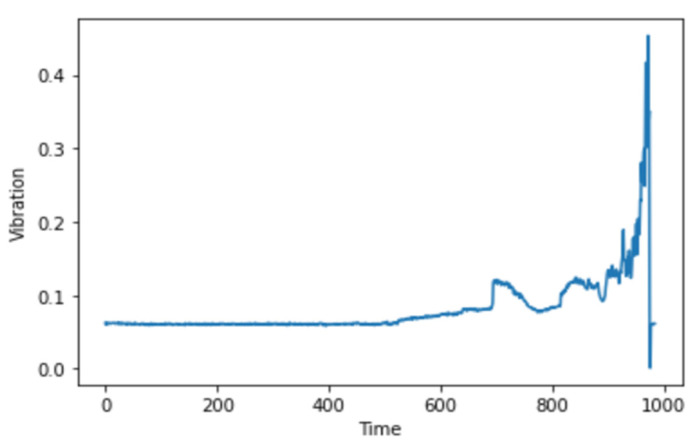
Vibrational signal of Bearing 1 through the entire experiment.

Anomaly detection and RUL prediction using spectrogram analysis and deep learning methodology is applied to the NASA bearing dataset Test 2 Bearing 1. The experimental results are explained in Figure 10:

### 4.1. K-Means Clustering

**Figure 11:**
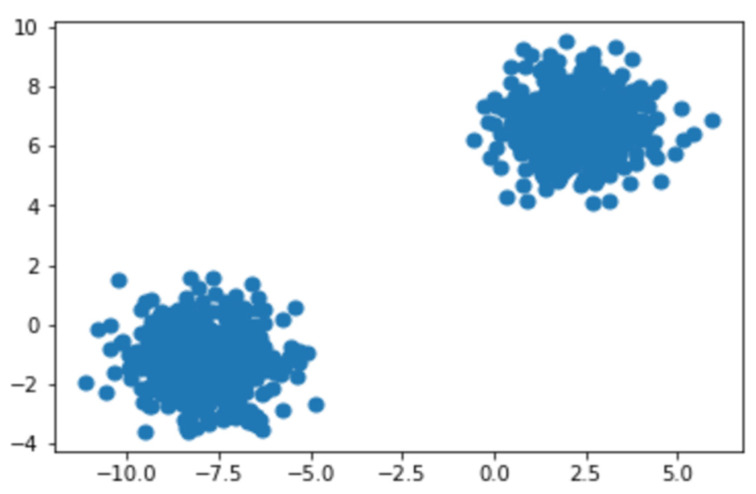
Clusters identified by K-means algorithm.

As the spectrogram images that were generated needed to be given as input for the convolutional neural network, which is a supervised learning algorithm, we needed annotated data [40]; hence the spectrograms were labeled on the basis of whether they depicted a normal vibrational signal or an abnormal vibrational signal. To label our data, we passed the spectrogram images generated before into a K-means clustering algorithm. The K-means algorithm separates a collection of N samples X into K disjoint clusters C, each of which is characterized by the cluster’s mean. Our K was taken as 2 so as to split the dataset into two clusters or groups indicating normal vibrational signals and abnormal vibrational signals. The two clusters can be seen in Figure 11.

As we know that the dataset is a “test-to-failure” experiment, we can conclude that, initially, the device was healthy and working. This means the group or cluster containing the first few images can be labeled as the healthy data points and healthy spectrograms, and the next cluster identifies the abnormal states of the device, and their spectrograms can be labeled as abnormal.

**Figure 12:**
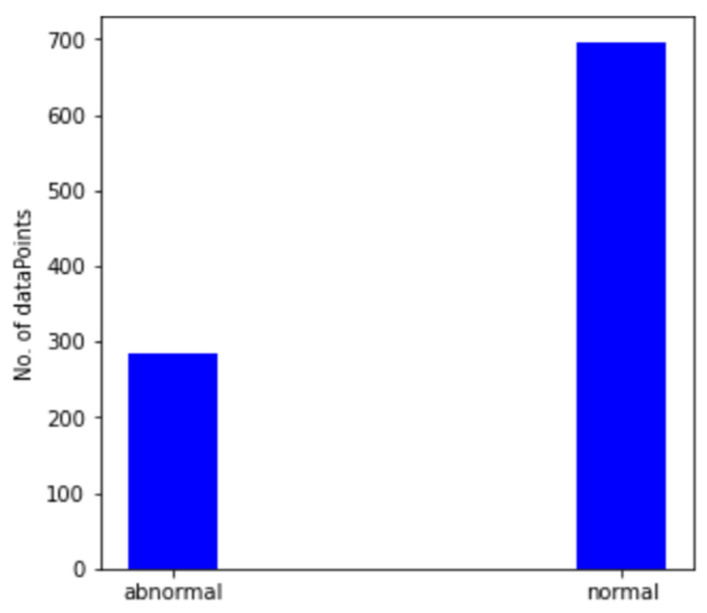
Count of normal vs. abnormal vibrational signals.

Figure 12 shows the output of our K-means data labeling system. It has now categorized and labeled spectrograms into two groups. The first group, or healthy group, contains 696 images, and the latter group, or abnormal state, contains 285 images. The images are automatically split into separate datasets (folders) to be fed into the CNN model.

### 4.2. Convolutional Neural Network

**Figure 13:**
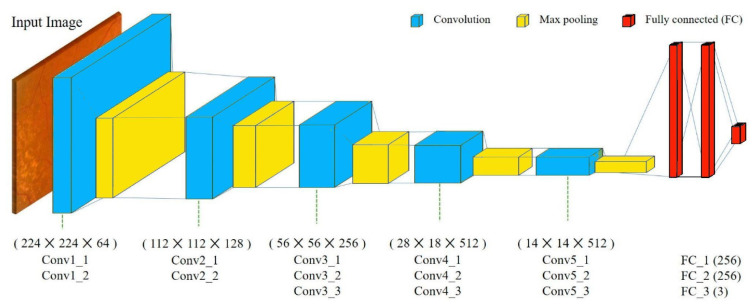
VGG-16 CNN model architecture.

Now that our spectrogram images are generated and labeled, we can now train our neural network on this data to teach it to predict whether the provided spectrogram depicts a healthy vibrational signal or an abnormal vibrational signal. As shown in Figure 13 First, the spectrogram image is pre-processed, i.e., which means the image of shape (224, 224) is converted into (224, 224, 3), where 3 is the number of channels, red, blue, and green, respectively. Then, without scaling, it will zero-center each color channel with respect to the ImageNet dataset. In addition, it appends our dataset into a numpy array making the input shape into (984, 224, 224, 3), where 984 is the number of samples. The images were trained against three different CNN architectures.

The dataset was divided into training and testing data, with 80% of the data being training data (786 images) and 20% for testing and validation (196 images) and a batch size of 32.

We used an Adam optimizer to change the attributes of the neural network, such as weights and learning rate, to reduce the losses. After testing with multiple values for the hyperparameters for adam, we used an initial learning rate (𝛼) as 0.0007, the exponential decay rate for the first moment estimates (beta1) as 0.9, the exponential decay rate for the second-moment estimates (beta2) as 0.999 and epsilon to prevent any division by zero in the implementation as 1e-08 to train the model.

#### 4.2.1. VGG16

On training the VGG-16 CNN model as shown in Figure 13 for 13 epochs, we could see the quick learning of our effective model as its validation loss reached 0.1002.

**Figure 14:**
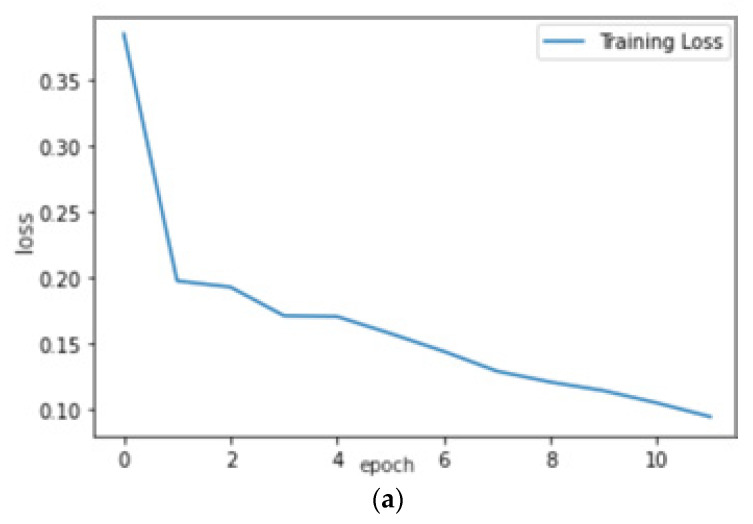

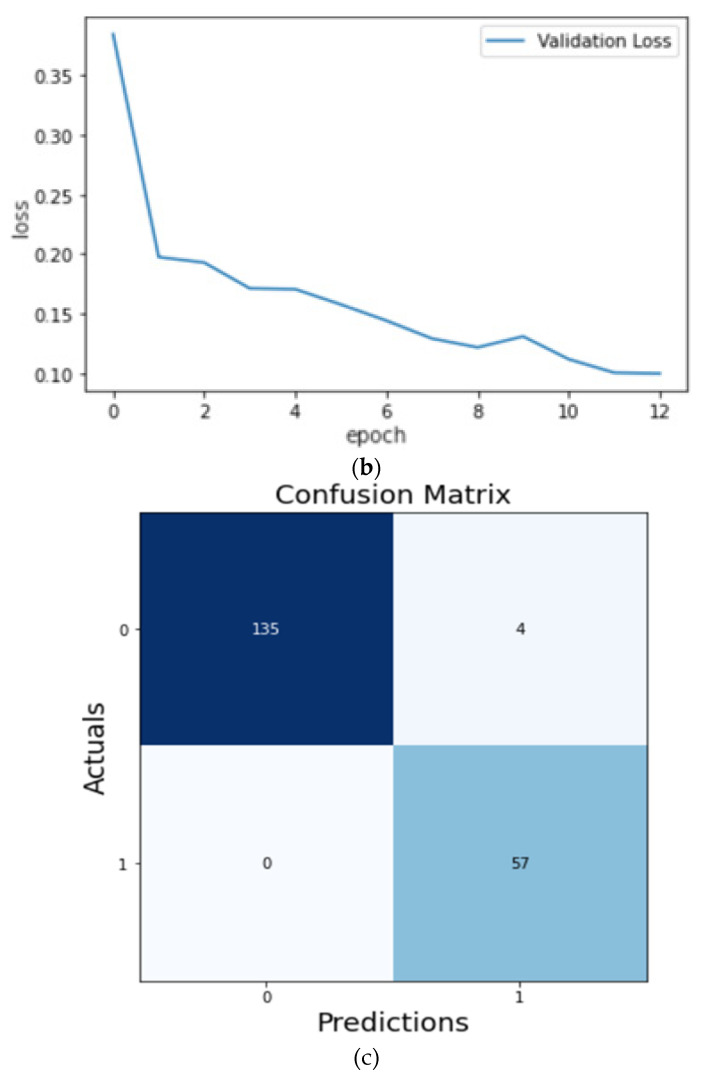
(**a**) Training loss curve, (**b**) validation loss curve, and (**c**) confusion matrix of validation data.

Figure 14c shows the confusion matrix of the predictions made on the testing data. We can see that 135 images were correctly predicted as normal signals (true positives), and 57 images were correctly predicted as abnormal signals (true negatives). After training our model, we have recorded our accuracy metric scores in Table 2.

#### 4.2.2. Inception V3

**Figure 15:**
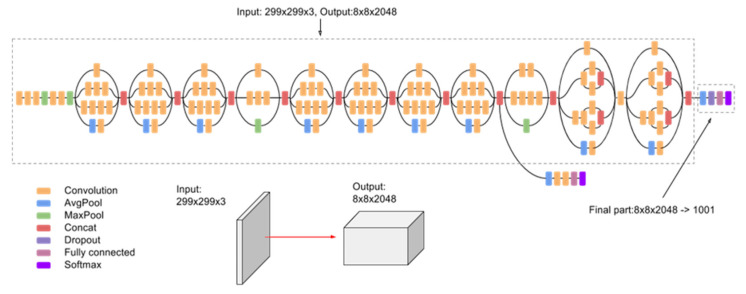
InceptionV3 CNN model architecture.

On training the InceptionV3 CNN model as shown in Figure 15 for 20 epochs, we could see the quick learning of our effective model as its validation loss reached below 0.1002.

**Figure 16:**
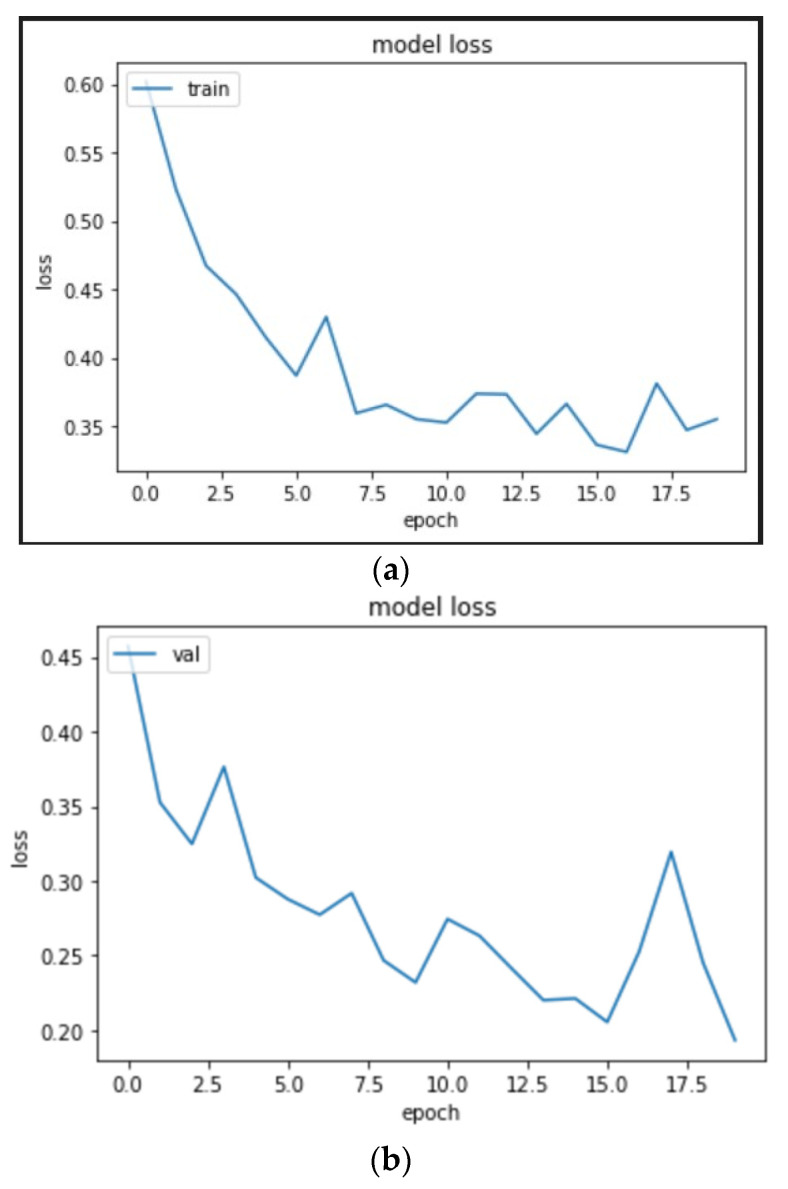

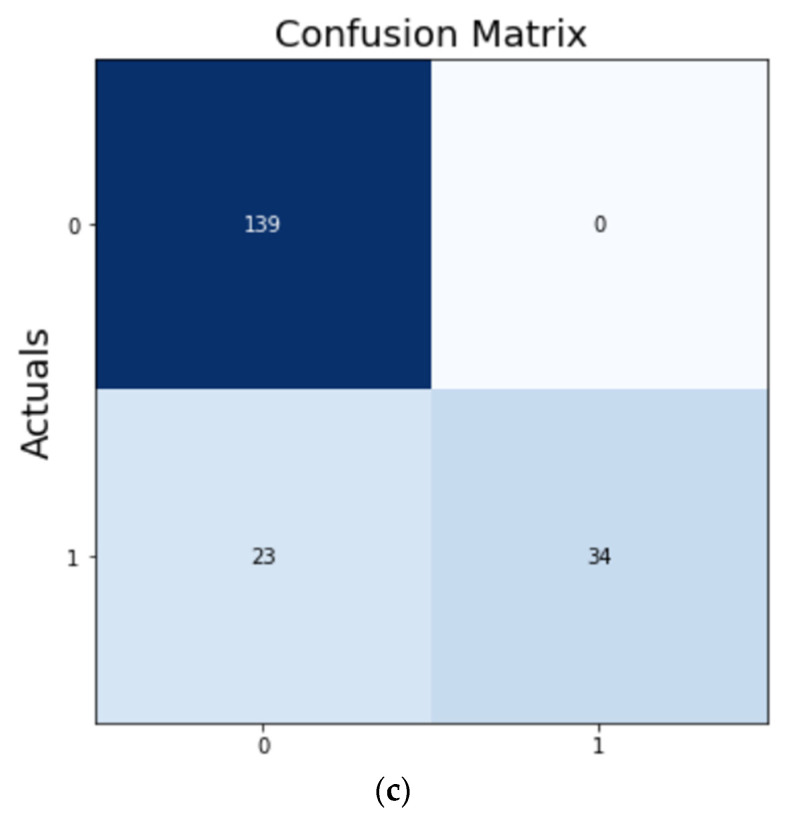
(**a**) Training loss curve, (**b**) validation loss curve, and (**c**) confusion matrix of validation data.

We can see from Figure 16 and Table 3 that while the InceptionV3 CNN model was able to predict 139 images correctly as normal signals (true positives) and 34 images correctly as abnormal signals (true negatives), the accuracy of the model fell short in comparison to the VGG-16 CNN model.

#### 4.2.3. ResNet 50

**Figure 17:**
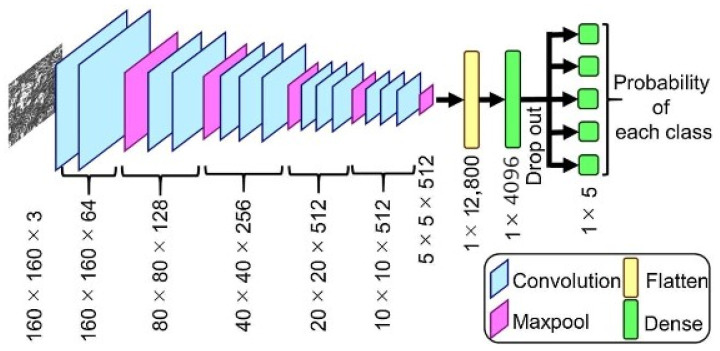
ResNet50 CNN model architecture.

On training the ResNet model as shown in Figure 17 for 19 epochs, we could see the quick learning of our model as its validation loss reached 0.3404.

**Figure 18:**
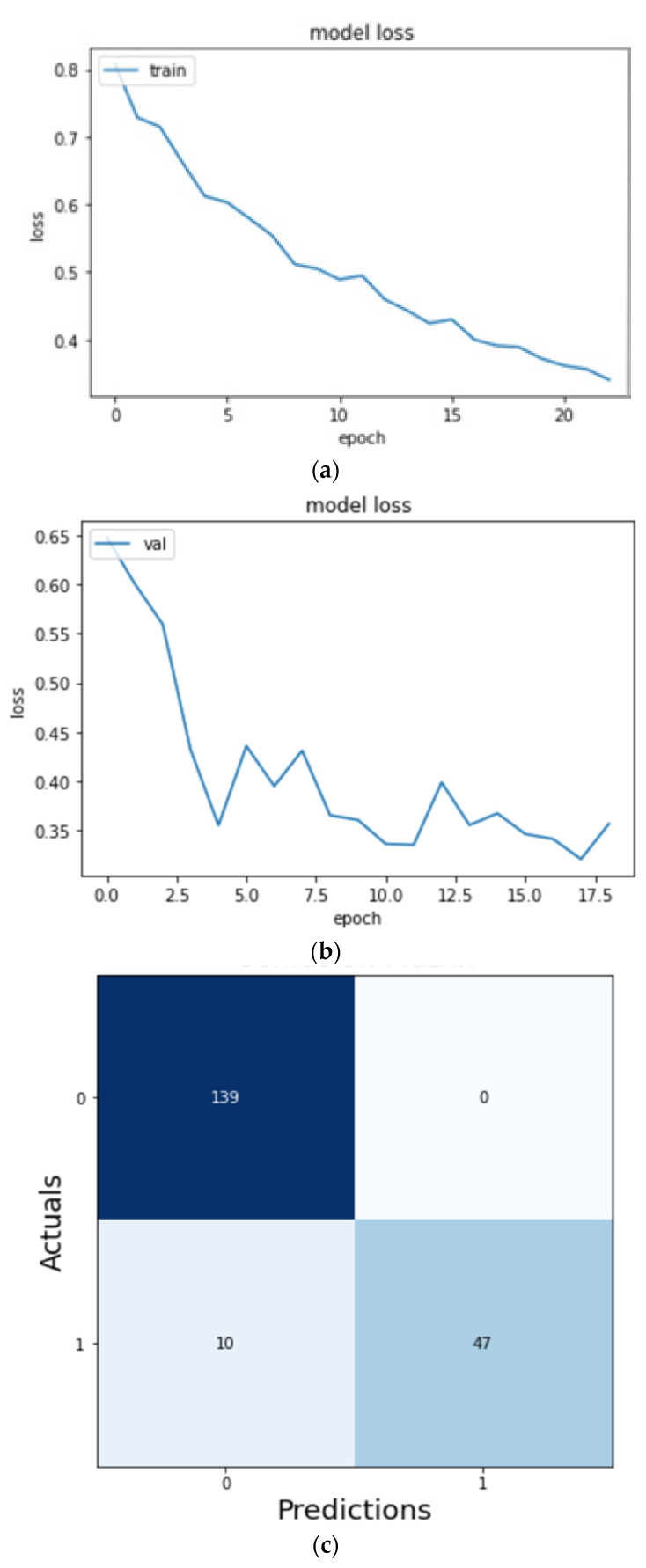
(**a**) Training loss curve, (**b**) validation loss curve, and (**c**) confusion matrix of validation data for ResNet50 model.

We can see from Figure 18 and Table 4 that while the ResNet50 CNN model was able to predict 139 images correctly as normal signals (true positives) and 47 images correctly as abnormal signals (true negatives) and performed better than the InceptionV3 CNN model, the accuracy of the model again fell short in comparison to the VGG-16 CNN model.

### 4.3. Explainable AI

Now that we have a working CNN model to accurately predict whether a given spectrogram image is healthy or abnormal, we can also train the model to identify what part of the image helped the model to make its prediction. We used a LIME (local interpretable model-agnostic explanations) image explainer to help interpret the working process of the CNN model. By turning on and off some superpixels in the image, LIME will build a pattern that looks similar to our input image. LIME will next use our trained model to estimate the class of each bogus data point created. If your input is a picture, this is where you will get a forecast for each scrambled image. The cosine distance between each noisy picture and the original image is determined if the input data is an image. The more similar the noisy image is to the original image, the more weight and value it carries. The linear regression model is then fitted using weighted fabricated data points as the last step. We will obtain the fit factor of each feature after this phase, exactly like in a standard linear regression analysis. Now, if we sort the coefficients, the characteristics with greater coefficients are the ones that have a significant impact on our CNN machine learning model’s prediction.

**Figure 19:**
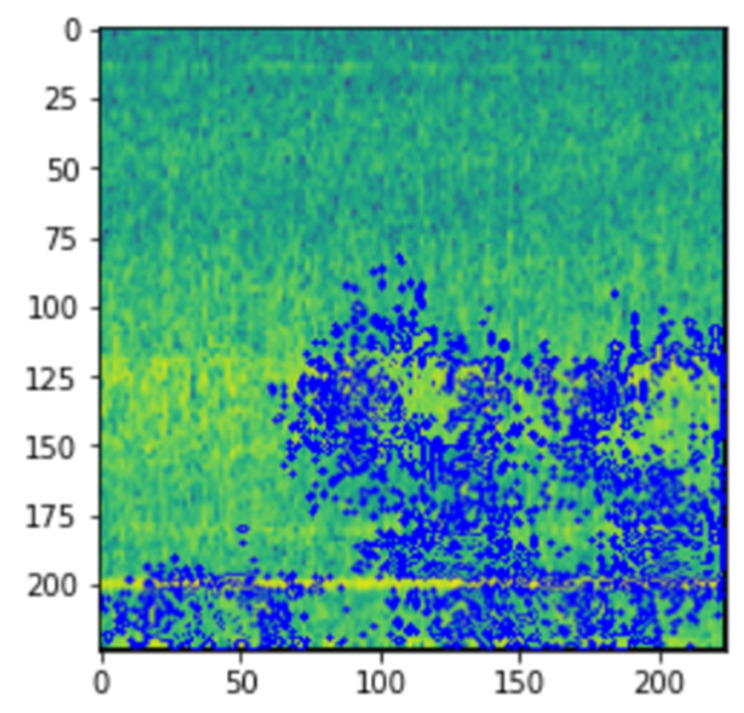
LIME image explainer on healthy vibrational signal.

**Figure 20:**
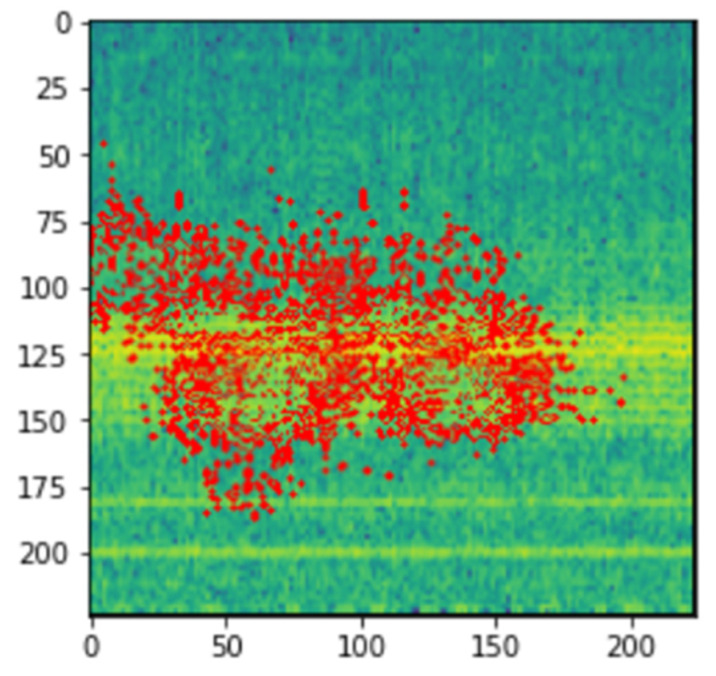
LIME image explainer on abnormal vibrational signal.

From Figure 19 and Figure 20, we can see the blue markings (healthy) and red markings (abnormal) on the images, which indicate that the features depicted in that area of the image help the model in making the prediction. We can see that the model is mostly focused on the areas that are colored yellow. This indicates that the model is able to understand that this represents some feature of the signal. As we had seen in the spectrogram generation subsection, this yellow region represents the amplitude of the signal. This tells us that the CNN model is able to automatically pick up on signal features from an image and use that information to classify those images without implicitly being told to. 

**Figure 21:**
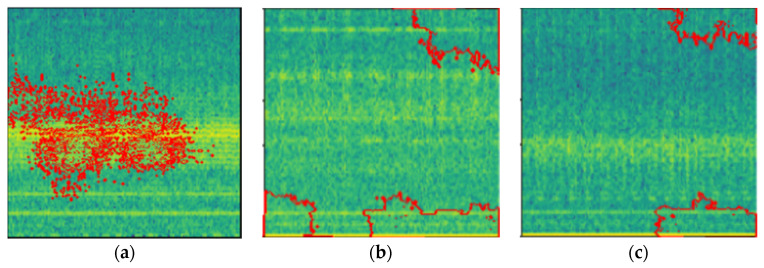
LIME explainer applied on various bearing defects (**a**) outer-race-defect, (**b**) inner-race-defect, (**c**) roller-defect.

The LIME explainer was then applied to various types of bearing faults so that we see how our model can differentiate between the various types of defects as shown in Figure 21. This helps us to obtain an insight into how the model can understand the different types of defects from the spectrogram images. The LIME explainer was applied to outer-race-defect, inner-race-defect, and roller-defect. The results are depicted below.

### 4.4. Anomaly Detection

Now that we have a trained image recognition model, we have passed the images in chronological order to the model and counted the number of images the model predicted as abnormal. A sliding window of size 1% of the dataset, i.e., 9.8~10 images, was taken. If all the images in the window are predicted as abnormal by the CNN model, then we can mark that window as an anomaly. The first anomaly is the first window that is detected, and we can assume that the machine/device is soon going to fault. If 10 windows in a row are marked as anomalies, then we can assume the device has faulted or an error has taken place.

**Figure 22:**
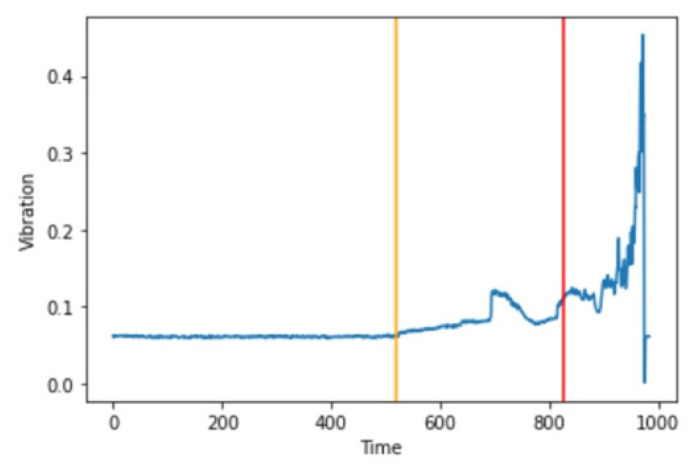
First anomaly and fault detection on Bearing 1.

In Figure 22, we can see that the yellow line at spectrogram #520 or 86.66 min from the start of the experiment is where the first anomaly occurred. The red line indicates that a fault has taken place, and our model predicts that spectrogram #827 or 137.83 min from the start of the experiment is where the fault has taken place.

### 4.5. Feature Engineering

To train a linear regression model for the RUL prediction, we need to extract some features from our vibrational signals. Working with raw vibrational signals was not the best approach we could take. After all, we are looking for a slow accumulation in a very dynamic signal. Conventional wisdom dictates applying signal processing techniques to waveforms, compressing, analyzing, and interpreting data, and extracting useful information for diagnostic and prognostic purposes. Traditionally, waveforms were analyzed by extracting characteristics in the time and frequency domains. More exactly: mean, standard deviation, skewness, kurtosis, entropy, root mean square, max, peak-peak values.

### 4.6. Remaining Useful Life Prediction

Every entry in the data frame is given a cycle number in chronological order. After extracting the features from the vibrational signals, the RUL column is appended to our new data. This column is counter with values consisting of the number of cycles remaining before a fault occurs. This means the RUL value at spectrogram #1 or cycle number 1 will be 827, as we had noted that to be the point of fault in the previous subsection. RUL value at spectrogram #(*n*) or cycle number *n* will be (827-*n*). 

**Figure 23:**
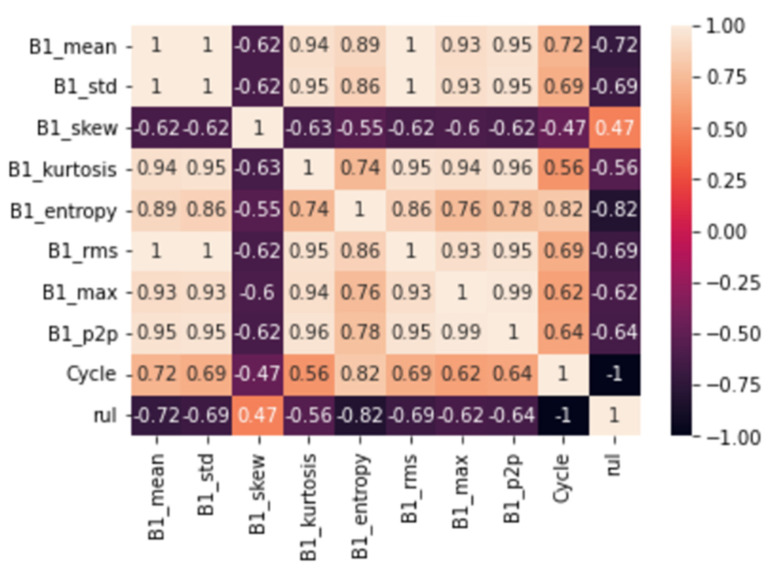
Correlation heatmap.

**Figure 24:**
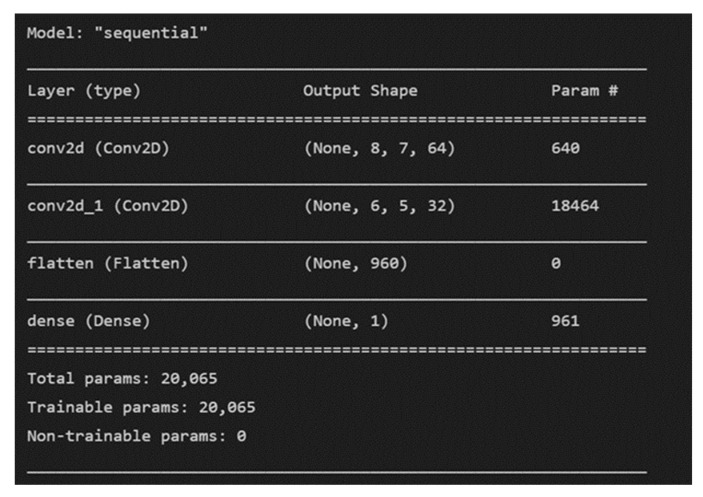
RUL prediction CNN model architecture.

Figure 23 shows how the various features extracted correlate with each other and the RUL of the device. These features, along with the RUL, are given as input into a simple CNN model with two convolutional layers as shown in Figure 24.

The CNN model is trained with a batch size of 64 for 150 epochs and gives an RMSE (root mean square error) of 10.71 as shown in Table 5.

**Figure 25:**
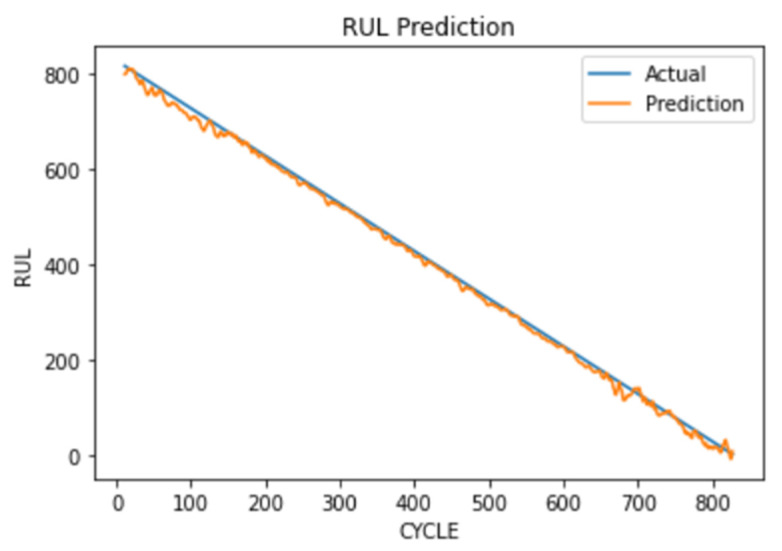
RUL actual vs. predicted value.

Figure 25 shows that with the trained RUL prediction CNN model, if we provide the model with how long it has been since the start of the reading, it can tell you the remaining useful life of the device.

**Figure 26:**
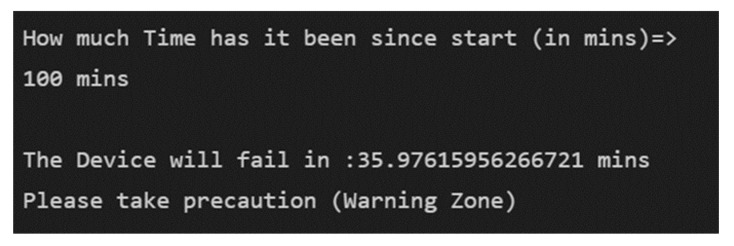
Framework tool output.

If we provide the tool with 10 vibrational snapshots, it will automatically calculate and extract the time features and predict the remaining useful life of the device accurately as shown in Figure 26.

## 5. Discussion

The objective of this study is to develop a model for the estimation of remaining useful life (RUL) using efficient algorithms. This model is designed and presented to avoid machine degradation at the right time to save time and resources, thereby increasing machine life and avoiding accidents [1,41,42]. Our framework uses effective deep learning models that are indeed known to have high intellectual capabilities and generate effective results. Compared to many studies, our proposed technique estimates RUL with less computational overhead, which increases its flexibility. The main contributions of this study include:

1. Generating spectrogram for better visual details and performing FFT analysis on the same and use of K-means clustering on an unsupervised learning algorithm to identify the unlabeled spectrogram images and clustering them;
2. Deep learning-based anomaly detection using VGG-16, InceptionV3, and ResNet, pretrained convolutional neural network models and obtaining satisfactory accuracy of 98.47%
3. Usage of explainable AI (LIME) for examining the CNN model;
4. Deep learning-based RUL prediction using the convolutional neural network model and obtaining an RMSE of 10.71.

## 6. Conclusions

With the advent of greater versions of the industry, predictive maintenance is growing with every coming day, and our model helps:

1. Saving money and time;
2. Reduce maintenance costs;
3. Make machinery usage safer and more reliable;
4. Maximize efficiency and increase equipment lifetime.

With the above features, our need for an all-delivering artificial intelligence framework, precisely in the field of predictive maintenance, is increasing and is required now more than ever. In the future, the work can be extended to implement the framework under various operational scenarios using the transfer learning approach.

## Figures and Tables

**Table 1:** Describes the test-to-failure experiment details for Tests 1 and 2.

	1st Test	2nd Test
Time Duration of the Recordings	22 October 2003 at 12:06:24 to 25 November 2003 at 23:39:56	12 February 2004 at 10:32:39 to 19 February 2004 at 06:22:39
No. of Files	2156	984
Interim	10 min (For the 43 files that were taken at the start of the experiment—every 5 min)	10 min
Failures Encountered	Inner race in the 3rd bearing and roller element in the 4th bearing	Outer race in the 1st bearing

**Table 2:** Accuracy metric readings of VGG-16.

Accuracy Metric	Score
Accuracy	98.4694%
Recall Score	0.9474
F1 Score	0.9729

**Table 3:** Accuracy metric readings of InceptionV3.

Accuracy Metric	Score
Accuracy	88.2653%
Recall Score	0.5964
F1 Score	0.7472

**Table 4:** Accuracy metric readings of ResNet50.

Accuracy Metric	Score
Accuracy	94.8980%
Recall Score	0.8245
F1 Score	0.903846

**Table 5:** RUL prediction CNN model accuracy metrics.

Metric	Value
MSE	114.70
RMSE	10.71
R2 Score	0.9734
